# The Effort Syndrome: Soldier's Heart

**Published:** 1919-08-30

**Authors:** 


### THE STUDY OF FATIGUE.

### The Effort Syndrome: Soldier's Heart.

In a paper read before the Medical Society of the County of Herkimer, N.Y., Dr. George Edward Barnes gives some most interesting comments, which attempt to explain the effort syndrome and the condition popularly known as soldier's heart. In the first place he urges that the effort syndrome is one of the many manifestations of neurasthenia, and in accordance with our lack of exact knowledge on this latter condition it would not, therefore, seem surprising that the true bearing of the cardiac irritability is but partially understood. Very truly the author suggests that if we realise that *all* people whom we treat have brains and minds, and that, whatever may be their natural endowment and intellectual state, they experience emotions, and that, unless they are abnormal, they are capable of suffering from severe affective diseases, then we must also realise the enormous importance of these states. In average practice not only might a certain percentage of the cases seen by doctors be classified as affective disturbances and neurasthenia, but these states undoubtedly constitute a greater or lesser complication in many other ailments. For no person can efficient and scientific treatment have been claimed unless due account is taken of these profoundly human and very prevalent conditions. It is from this standpoint, therefore, that Dr. Barnes attempts to give us a summarised account of his explanation and treatment of the effort syndrome, and, although our own experience may not lead to a perfect sympathy with his views, yet there are some very valuable ideas expressed.

### An Explanation.

The parts principally involved are the so-called "sympathetic nervous system," and those portions of the brain concerned in the development and propagation of violent affective currents. Also we may include, from the standpoint of the fully developed disease, most of the functioning organs of the body, chiefly, perhaps, the arteries, heart, stomach, and intestines.

The affective portions of the brain and the sympathetic nervous system are excessively irritable to all stimuli, as is so evident—*e.g.*, in the use of certain drugs, as epinephrin (adrenalin) and cocaine. The pathologico-physiological forces that are active are stimulating currents originating in the "violent" affective centres of the brain and in the structures of the sympathetic nervous system. The signs and symptoms of the disease are caused *directly* by these pathological currents stimulating to excessive activity certain organs, particularly the heart and arteries, and *indirectly* by the effect which this excessive activity has on other organs

A classification into three forms is given: (1) Primary. (2) Secondary or reactionary, which is subdivided into (*a*) that following nervous states of emotion and (*b*) that following severe constitutional disease. In the instance of the first group violent affective currents are aroused by certain ideas, with or without physical exertion, and, passing through the sympathetic system, these cause certain activities in the viscera, with corresponding secondary results. It is obvious that corresponding effects would follow such ideas in a normal person, but it is suggested that in this disease there have been such oft-repeated, prolonged and gross affective stimuli passing in the same channels that the parts involved have become weakened, and therefore excessively irritable. In Group 2 (*a*) a different mechanism is put forward. Prior to the development of the disease "pathetic affectivity" dominates the whole being, even to the extent of complete nervous prostration. As recovery commences pathetic affectivity subsides, and the hitherto completely suppressed sympathetic system and the affective currents from the brain spring into ready activity which may become excessive. Essentially this is a rebound from nervous prostration. With subdivision (*b*) there is a similar reaction, but arising upon a nervous system weakened by constitutional disease or direct infection.

Such is but a poor extract and a rough indication of the author's essential points on the nature of effort syndrome and the way in which it may arise.

A suggestion is also made that a condition exactly comparable with the effort syndrome appears in many patients about to undergo operation, and that this may even constitute one of the two chief forms of surgical shock.

### The Treatment.

The essential aim is to subdue and regulate the pathological activities already outlined. Psychotherapy must be used with judgment according to indication. In the primary group it reaches its maximum importance, for here the patient's own thoughts and emotions are the immediate cause. In the second the same measures may be called for, since, although the disturbing affective currents are *causative*, and yet they do not originate immediately from the thoughts of the patients, still such a patient is usually so much and so unduly concerned about his own condition and everything in general that psychotherapy is necessary. Actually a combination of the primary and secondary forms is most commonly met with. Turning to general measures, we remember that rest, bodily and mental, is essential. Carefully graduated, controlled exercise may be indicated; and, indeed, it may be said that with these patients it is often difficult for any real rest to be taken, because hyper-activity of the sympathetic system so stimulates the organs of the body that rest is absolutely impossible unless proper medication is employed. In the author's experience some cases require hypnotics. Occasional doses of vasodilators are needed in all cases. Iodides and thyroid extract are particularly useful with the secondary type, while digitalis and strophanthus have their own

**The Study of Fatigue—(*continued*).**

indications. One risk in medication is that to produce the required effect on one organ the dose may have a greater effect than is desired on another:

However, medicinal treatment is most essential for proper recovery, and in severe cases it is really life saving. It will, too, prevent some cases from being sent to the asylum under an erroneous diagnosis. These patients are far from insane, if one knows what insanity is.

In conclusion, apart from diet regulated on general principles, Dr. Barnes summarises that the residual hyperchlorhydria frequently encountered must be relieved, the body kept amply warm, and the treatment directed towards general improvement of the central nervous system. The length of treatment with the primary group is sometimes reasonably short, but in cases of the secondary group, especially those that have been prostrated, it may even require many years. But it is cheering to read the final result of his experience. Prognosis, he says, is, contrary to general belief, excellent if proper treatment be intelligently employed.

